# Successful treatment of hairy cell leukaemia with pegylated interferon-alpha-2A

**DOI:** 10.3389/pore.2025.1612108

**Published:** 2025-05-21

**Authors:** Kata Ferenczi, Zsófia Flóra Nagy, Ildikó Istenes, Hanna Eid, Csaba Bödör, Botond Timár, Judit Demeter

**Affiliations:** ^1^ Department of Internal Medicine and Oncology, Semmelweis University, Budapest, Hungary; ^2^ Department of Pathology and Experimental Cancer Research, Semmelweis University, Budapest, Hungary

**Keywords:** hairy cell leukemia, interferon, HCL, pegylated interferon-alpha, severe infection

## Abstract

**Introduction:**

Hairy cell leukemia (HCL) is an indolent B-cell lymphoproliferative disease. Interferon-alpha (IFN-alpha) was the first successfully used drug in HCL; its favourable effect has been known since the early 1980s. However, currently the first-line treatment of the disease consists of purine nucleoside analogs.

**Objectives:**

The aim of our study was to assess the efficacy of pegylated IFN-alpha in HCL patients treated with this drug at a single university center.

**Methods:**

We report the treatment characteristics and outcome of seven classical HCL patients treated with pegylated IFN-alpha at the Department of Internal Medicine and Oncology, Semmelweis University.

**Results:**

As a result of pegylated interferon-alpha treatment, 3 of 7 patients (3/7) achieved an unconfirmed complete remission, 3 of 7 patients (3/7) achieved partial remission. One patient had stable disease while receiving pegylated IFN-alpha. Only mild adverse effects and no infectious complications were observed during our treatment.

**Conclusion:**

Our clinical data support that pegylated IFN-alpha in monotherapy is effective and safe even in elderly and frail HCL patients. It may also be a preferred therapeutic option in patients with profound immunosuppression and in patients with severe active infections.

## Introduction

Hairy cell leukemia (HCL) is an indolent B-cell lymphoproliferative disease characterized by pancytopenia, splenomegaly, and infiltration of bone marrow, liver, and spleen with mature B cells. HCL is a rare neoplasm representing 2% of lymphomas [1, 2]. The disease is well characterized based on cytomorphologic and immunophenotypic features, and its defining parameters as a distinct entity have remained unchanged since the 2001 WHO classification [3].

Common symptoms arise as a consequence of cytopaenias and splenomegaly [4]. Infectious complications are common, due to both the underlying immunosuppression from granulocytopenia and monocytopenia and decreased activity of NK cells and dendritic cells [3].

The diagnosis is based on the histological examination of the bone marrow trephine biopsy specimen [5]. The well-established surface antigen expression pattern (CD20^+^ CD103+ CD25^+^) were complemented by a genetic marker in 2011 thanks to the groundbreaking discovery of the p.V600E mutation in the *BRAF* gene characteristic of this entity [6].

The current gold standard first-line treatment of hairy cell leukemia is the administration of purine nucleoside analogs (cladribine or pentostatin) [7]. Purine nucleoside analogues are highly immunosuppressive, thus ongoing infections in HCL patients limit their usability [8]. Furthermore, during the COVID-19 pandemic BRAF inhibitors came into play as an alternative therapy since they do not cause neutropaenia [9]. Since this indication is off-label for BRAF inhibitors and they are not available worldwide, the search for a new alternative has begun. Recombinant Interferon-alpha is recommended as a first-line treatment in pregnancy, in patients with severe neutropenia or in those who are not eligible for purine nucleoside analogues [10]. At the end of 2019 the production of both types of recombinant interferon-alpha was discontinued worldwide, which coincided with the COVID-19 pandemic and created a crisis situation in this regard. Thus, the administration of pegylated interferon-alpha (peginterferon alfa-2a) (PEG-IFN) became available in Hungary as a possible alternative.

## Patients and methods

During our analysis we complied with the Helsinki declaration and its later amendments. All participants gave their informed consent to the analysis of the data. The Medical Research Council has granted the study its approval under the ethical number 28564-2/2017/EKU.

A total of 7 HCL patients with classical phenotype (4 males, 3 females) received pegylated interferon-alpha treatment at the Department of Internal Medicine and Oncology, Semmelweis University. These patients have been treated previously (between 2016 and 2020) with recombinant interferon-alpha, except for 3 patients who received pegylated interferon-alpha as a first line treatment. Pegylated interferon-alpha became available from the early months of 2020. Our follow-up period ended on October 31st of 2024. The median age at diagnosis was 67 years (range: 48–73 years, mean: 63.71 years), and at initiation of pegylated interferon-alpha treatment was 69,5 years (range: 59–75 years, mean: 69.14 years). The median time from diagnosis to PEG-IFN treatment was 24 months (range, 1–168 months). Patient characteristics are shown in Table 1.

**TABLE 1:** Patient characteristics of our cohort.

Patient	Year of birth	Sex	Age at HCL diagnosis	Comorbidities
Patient 1	1961	male	48	hypertension, mesenteric thrombosis, type 2 diabetes mellitus, umbilical hernia
Patient 2	1950	female	63	hiatus hernia, gonarthrosis, hypertension, thyreoiditis, postmenopausal osteoporosis
Patient 3	1946	female	72	liver cysts, uterine myoma, hypertension, angina pectoris
Patient 4	1949	male	71	nephrolithiasis, hypertension, hypercholesterolaemia, type 2 diabetes mellitus, GERD, hiatus hernia, basal-cell carcinoma, benign prostate hyperplasia,
Patient 5	1952	male	55	tonsillectomy, type 2 diabetes mellitus, gonarthrosis, chronic cholecystitis, hypertension, dilatative cardiomyopathy, amputation of both legs below the knee due to diabetes complications
Patient 6	1957	female	64	insulin resistance
Patient 7	1947	male	73	nephrolithiasis, lumboischialgia, prostate cancer, glaucoma, coxarthrosis, rectal cancer

Our diagnostic workup consisted of taking a detailed past medical history, a thorough physical examination, peripheral blood counts and flow cytometric evaluations according to standard procedures. The ultimate diagnostic procedure was the histological examination of the bone marrow biopsy sample. *BRAF* status was determined by immunohistochemistry, while the sample of one of the patient a had been studied by polymerase chain reaction and pyrosequencing of the product and in the this patient. In the later course of disease, *BRAF* positivity was proven by immunohistochemistry. Detailed description of *BRAF* V600E mutation–specific immunohistochemistry and the molecular analysis for *BRAF* mutations can be found in our 2023 publication [9].

When suspecting a relapse, we performed these examinations including BRAF immunohistochemistry again to confirm the relapse of the hairy cell leukaemia.

Clinical data were collected at regular follow-up visits and by medical chart review. Responses were evaluated based on standard criteria as published by Bohn *et al* [11]. A response was categorized as an unconfirmed complete remission (CRu) if the blood counts normalized and organomegaly resolved but no bone marrow biopsy was carried out to confirm the complete remission. Partial remission (PR) was defined as more than 50% improvement in cell counts, shrinking organ sizes and an at least 50% decrease in bone marrow infiltration as judged by the pathologist. Stable disease was defined when no significant changes in blood counts or organomegaly were observed [11].

## Results

At our clinic we have treated seven classical HCL patients with pegylated interferon-alpha between July of 2016 and October 31st of 2024 (interferon-alpha treatment until early months of 2020 and pegylated interferon-alpha from then on). Our mean follow-up time from first referral to our center was 92.14 months (range: 32–208 months). As previous treatment lines, one patient was heavily pretreated, receiving PEG-IFN-alpha in 6th line, one further patient received PEG-IFN-alpha in 4th line. In three patients pegylated IFN-alpha was administered as first-line treatment. In these patients excessive bone marrow infiltration was present. In 3 patients the pegylated IFN-alpha treatment was still ongoing at the time of analysis. Results of the pegylated IFN-alpha treatment in hairy cell leukaemia patients can be seen in Table 2.

**TABLE 2:** Results and characteristics of the pegylated IFN-alpha treatment in hairy cell leukaemia patients.

Patient	Therapy start	Dose	Previous therapy lines	Response to therapy	Adverse events	Therapy discontinuation	Duration of PEG-IFN-alpha treatment	Duration of PEG-IFN-alpha treatment response	Current therapy	Duration of follow-up from first referral to the center
Patient 1	2016.07.15 (interferon alpha), 2020.08.24 (pegylated IFN-alpha)	90 µg weekly	1st line: interferon alpha for several months, 2nd line: cladribine, 3rd line: one time rituximab infusion, 4th line: vemurafenib, 5th line: interferon-alpha, 6th line: PEG-IFN-alpha	CRu	liver enzyme elevation, nausea	due to liver enzyme elevation in the fall of 2022	26 months	11 months	venetoclax 400 mg/die	178 months
Patient 2	2022.02.22	45 µg weekly	1st line: PEG-IFN-alpha	PR	none reported	April of 2022, due to next treatment line initiation (cladribine)	2 months	1,8 months	no hematological therapy needed	32 months
Patient 3	2018.08.22 (interferon alpha), 2021.07.16 (pegylated IFN-alpha)	45 µg weekly	1st line: interferon alpha, 2nd line: PEG-IFN-alpha	CRu	none reported	ongoing pegylated IFN-alpha therapy	39 months	39 months	ongoing pegylated IFN-alpha therapy	74 months
Patient 4	2020.01.04 (interferon alpha), 2020.04.05 (pegylated IFN-alpha)	90 µg weekly and from February of 2022 45 µg weekly	1st line: interferon alpha, 2nd line: PEG-IFN-alpha	CRu	none reported	March of 2023, due to complete remission	35 months	54 months	no hematological therapy needed	57 months
Patient 5	2007-2010 (interferon alpha), 2020.12.23 (pegylated IFN-alpha)	90 µg weekly and from February of 2023 45 µg weekly	1st line: interferon alpha, 2nd line: cladribine, 3rd line: rituximab infusions 4 times, 4th line: PEG-IFN-alpha	PR	none reported	ongoing pegylated IFN-alpha therapy	47 months	47 months	ongoing pegylated IFN-alpha therapy	208 months
Patient 6	2021.02.04 (pegylated IFN-alpha)	45 µg weekly due to severe granulopaenia, then 90 µg weekly from 12th of April 2021 on	1st line: PEG-IFN-alpha	stable disease	flatulence	in July of 2021 due to next treatment line initiation (cladribine)	5 months	4,8 months	no hematological therapy needed	46 months
Patient 7	2021.01.14 (pegylated IFN-alpha)	90 µg weekly	1st line: PEG-IFN-alpha	PR	none reported	ongoing pegylated IFN-alpha therapy	45 months	45 months	ongoing pegylated IFN-alpha therapy	50 months

CRu, unconfirmed complete remission. PR, partial remission.

**FIGURE 1:**
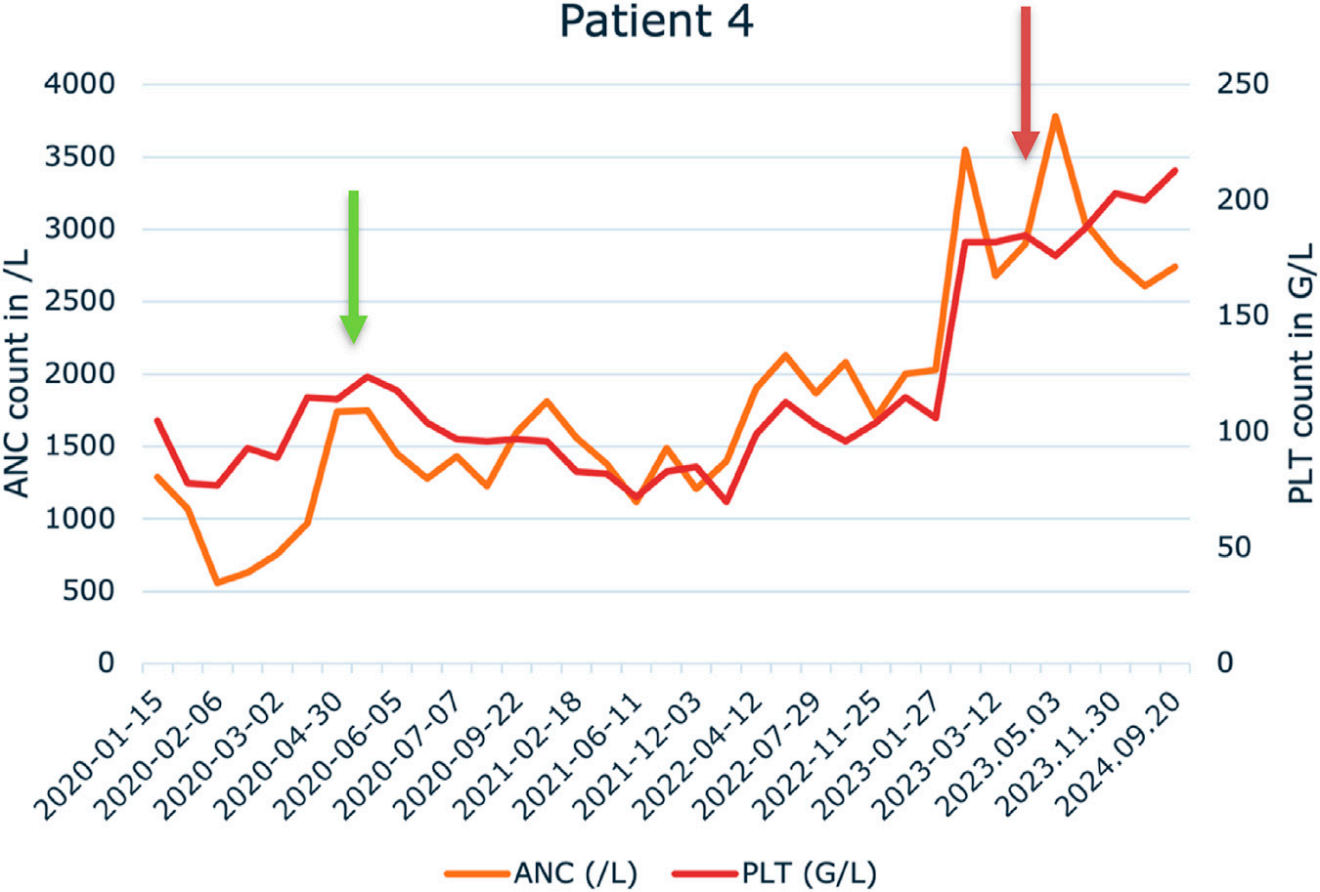
Change in absolute neutrophile count (ANC) and platelet (PLT) levels during the pegylated IFN-alpha treatment in Patient 4. Green arrow indicates the start of the treatment and red arrow indicates the cessation of the pegylated IFN-alpha therapy.

We administered IFN-alpha in pegylated formulation weekly (45–90 µg/week) by subcutaneous injection. As a result of treatment with pegylated interferon-alpha, 3 of 7 patients (3/7) achieved an unconfirmed complete remission, 3 of 7 patients (3/7) achieved partial remission. One patient had stable disease while receiving pegylated interferon-alpha. The detailed absolute neutrophil count (ANC) and platelet counts (PLT) can be seen in the Supplementary Material for each patient. An example is shown in Figure 1 for the case of patient 4.

No drug-related reactions or infectious complications were observed during our treatment. Three patients who received pegylated IFN-alpha once a week on a continous basis achieved partial remission. In the case of two patients who received cladribine treatment after pegylated IFN-alpha, after the completion of cladribine treatment hairy cells were not detected in the bone marrow anymore. During this follow-up time all patients are still alive. During our follow-up of Patient 7, we encountered a p16 positive squamous cell carcinoma of the anal canal 28 months after pegylated interferon-alpha treatment has started. In his past medical history a prostate adenocarcinoma is known, he underwent surgery for that in 2014, 7 years prior to starting PEG-IFN therapy.

## Discussion

The current gold standard first-line treatment of hairy cell leukemia is the administration of purine nucleoside analogs (PNA), such as cladribine or pentostatin [7]. Thanks to PNA treatment, the life expectancy of patients today does not differ from the life expectancy of the average healthy population, but 40% still have a relapse [12, 13]. While purine analogues are effective and safe in middle aged or younger and fit patients, in the treatment of older and unfit patients PNAs yield an unsatisfactory response [10].

The clinical activity of IFN-alpha in HCL patients has been known since the early 1980s. IFN induced responses were partial and of relatively short duration and most patients eventually relapsed after discontinuing treatment, with a median time to treatment failure ranging from 6 to 25 months [14]. It has been also reported, that long term maintenance IFN-alpha therapy after a short induction period can lead to longer remission periods and durable response [15, 16]. Also, in treatment naïve patients minimal doses of IFN-alpha seem to be effective [17]. In our cohort we did not observe a serious side effect leading to treatment discontinuation, which also fits the literature data [18].

Our results are in good concordance with the literature data. A 30 years retrospective analysis from Italy has found that IFN-alpha treatment of 74 HCL patients yielded a favorable response in almost all patients and even CR was achieved in 24% of patients [19]. This comprehensive study also highlights 3 key areas of use for IFN-alpha: patients with comorbidities that limit therapeutic agents, elderly patients, and times of a serious pandemic (due to its immunomodulant rather than immunosuppressive properties) [19–21]. Also, as we have seen in over the course of the COVID-19, that IFN-alpha is suggested by international consensus guidelines over PNAs, since it does not cause a drop in neutrophil counts thus does not elevate the risk of infection in these patients [20].

In the 1990-s the occurrence of secondary neoplasms in HCL patients treated with IFN have been first described. Contradicting reports have been published on the topic, an Italian group did not find an elevated risk of secondary neoplasms among HCl patients, while in a large cohort an increased incidence of secondary tumors, especially Hodgkin and non-Hodgkin lymphomas was observed [22, 23]. Our group also reported on the development of diffuse large B-cell lymphoma in two HCL patients a long time after their HCL diagnosis [24]. Our results from our small patient cohort do not allow to draw any conclusion in this respect.

Dosing of pegylated interferon-alpha treatment in our opinion should start with 45 µg weekly since neutrophil numbers may still be dropping at the dose of 45 µg weekly (as seen in the supplementary figure in case of Patient 2), which is crucial in an ongoing infection or in a severely immunocompromised state. We hypothesize that by administering a dose of 90 µg weekly the granulocyte drop would be even more pronounced. We advise starting the treatment with 45 µg weekly and slowly titrating upward to the effective dose (usually 90 µg weekly).

Only two case reports have been published until now showing the efficacy of PEG-IFN-alpha HCL. In April 2022 *Furlan et al.*, reported on a 73-year-old maleimmunosuppressed HCL patient with *Mycobacterium* subsp. massilliense abscess. Due to the ongoing infection and his severely immunocompromised status his HCL disease was treated with a combination of vemurafenib and rituximab after a “priming” phase with PEG-IFN-alpha as a first-line treatment (starting dose of 135 µg weekly). However, vemurafenib had to be discontinued after only 4 days of treatment due to QT interval prolongation and the possibility of cardiac toxicity. After the discontinuation of vemurafenib, PEG IFNα-2a was administered alongside rituximab again until the resolution of the infection. Two months after the first rituximab infusion a control bone marrow trephine biopsy was carried out which proved a complete response to the treatment [25]. This treatment combination might be a potential therapeutic option in patients with active severe infection and for whom the use of purine nucleoside analogues (PNA) is contraindicated [25]. In December 2022 *De Novellis et al.*, reported an old and frail HCL patient treated with pegylated IFN-alpha in monotherapy (90 µg once a week) as first-line treatment. The patient had severe neutropenia. After 6 months of therapy the patient achieved complete remission [26].

IFN-alpha binds to cell surface receptors with two particular subunits: IFN-α receptor-1 and IFN-α receptor-2 and activates the JAK/STAT signaling pathways and leads to the expression of antiproliferative and proapoptotic genes [27–29]. Moreover, it can also inhibit hematopoietic stem cell growth [30]. These effects reduce leukemic cell growth and cause myelosuppression [27, 28, 30]. The administration of IFN-alpha in pegylated form before and during rituximab treatment could be effective because IFN-alpha can increase CD20 antigen expression on hairy cells and simultaneously stimulate CD8 T cell and NK cytotoxic activity and induce neoplastic clone suppression [25].

Our data also support that pegylated IFN-alpha monotherapy is effective and safe in old and frail HCL patients. It can also be a preferred therapeutic option in patients with profound immunosuppression and in patients with severe active infections. In the treatment course we suggest monitoring the complete blood counts, liver function tests and clinical chemistry values.

PEG-IFN is not recommended for patients with autoimmune disorders, as the development of autoantibodies and autoimmune disorders has been reported during treatment with alfa interferons, according to the summary of product characteristics. Patients predisposed to the development of autoimmune disorders may be at increased risk. Patients with signs or symptoms consistent with autoimmune disorders should be evaluated carefully, and the benefit-risk of continued interferon therapy should be re-assessed (accessed on the 3rd of January 2025^1^). Premedication (e.g., acetaminophen) prior to pegylated IFN-alpha treatment may reduce flu-like effects.

## Conclusion

In our current work, we present data and clinical outcomes of 7 patients diagnosed with hairy cell leukaemia who received pegylated interferon-alpha treatment at our tertiary center between 2016 and 2024. Our analysis shows that pegylated interferon-alpha is a safe and effective treatment even in elderly and frail HCL patients who suffer from severe cytopaenias and/or from ongoing infections. Our relatively long follow-up confirmed the efficacy of this treatment. Moreover, 3 of the patients are in an unconfirmed complete remission with no current need for hematological therapy, 3 more patients achieved partial remission and are on long-term pegylated interferon-alpha injections without notable side effects.

Overall, we recommend that the administration of pegylated interferon-alpha be considered in selected cases of hairy cell leukaemia either as first line treatment or in further lines, depending on the clinical situation.

## Data Availability

The datasets presented in this article are not readily available because data was analyzed from medical files which contain private information.

## Footnotes

1
https://www.ema.europa.eu/en/documents/product-information/pegasys-epar-product-information_en.pdf
