# Supplementary figures and images for: Successful treatment of hairy cell leukaemia with pegylated interferon-alpha-2A
Source: Pathol Oncol Res. 2025 May 21;31:1612108. doi: 10.3389/pore.2025.1612108 (PMC12134567; doi:10.3389/pore.2025.1612108)

Supplementary file

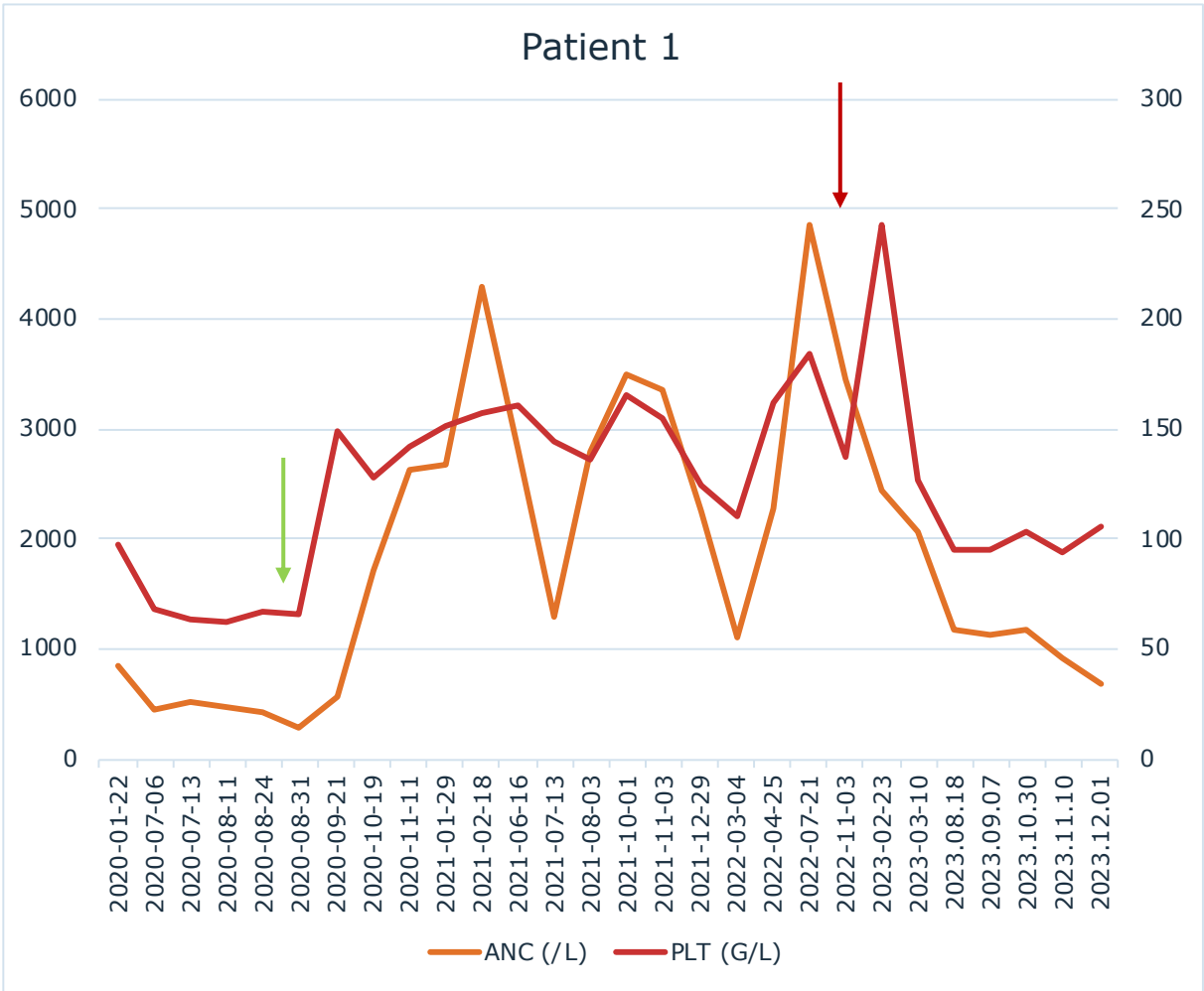

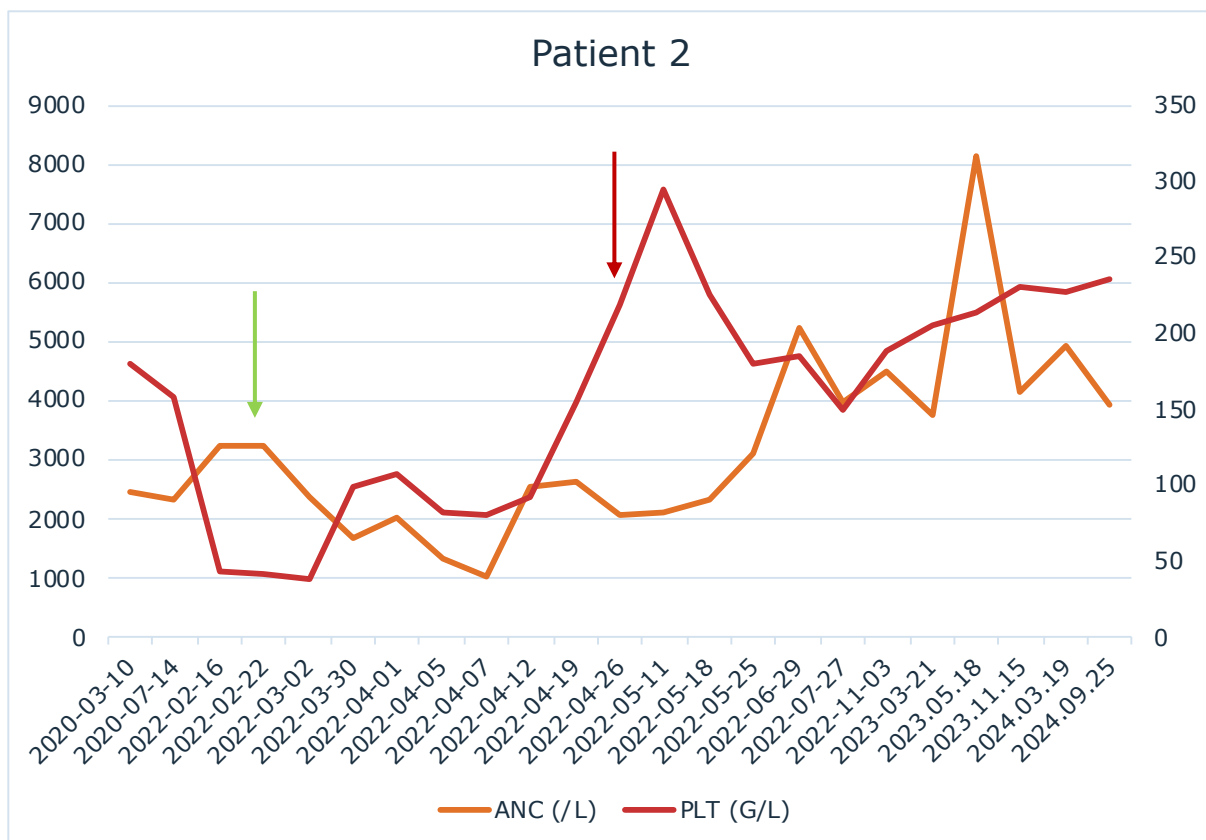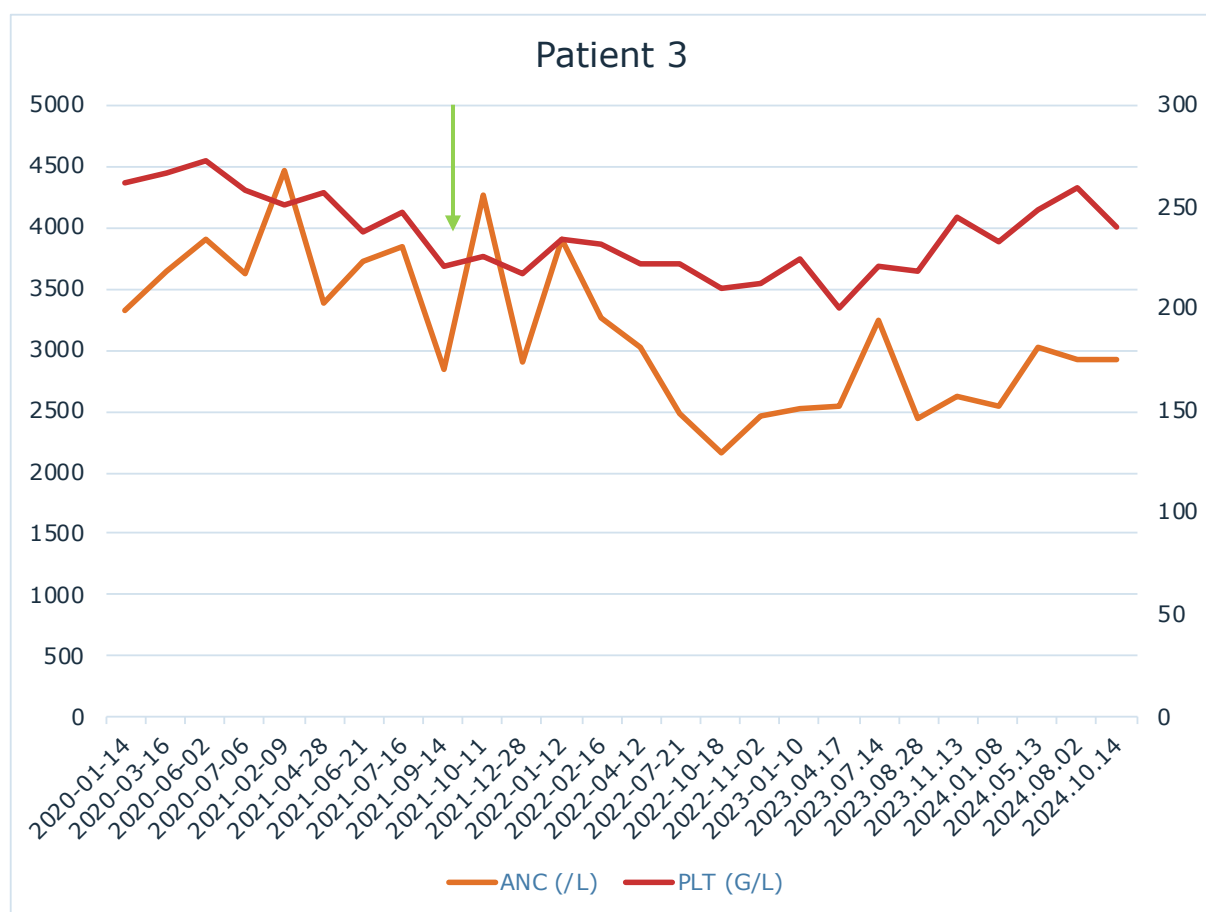

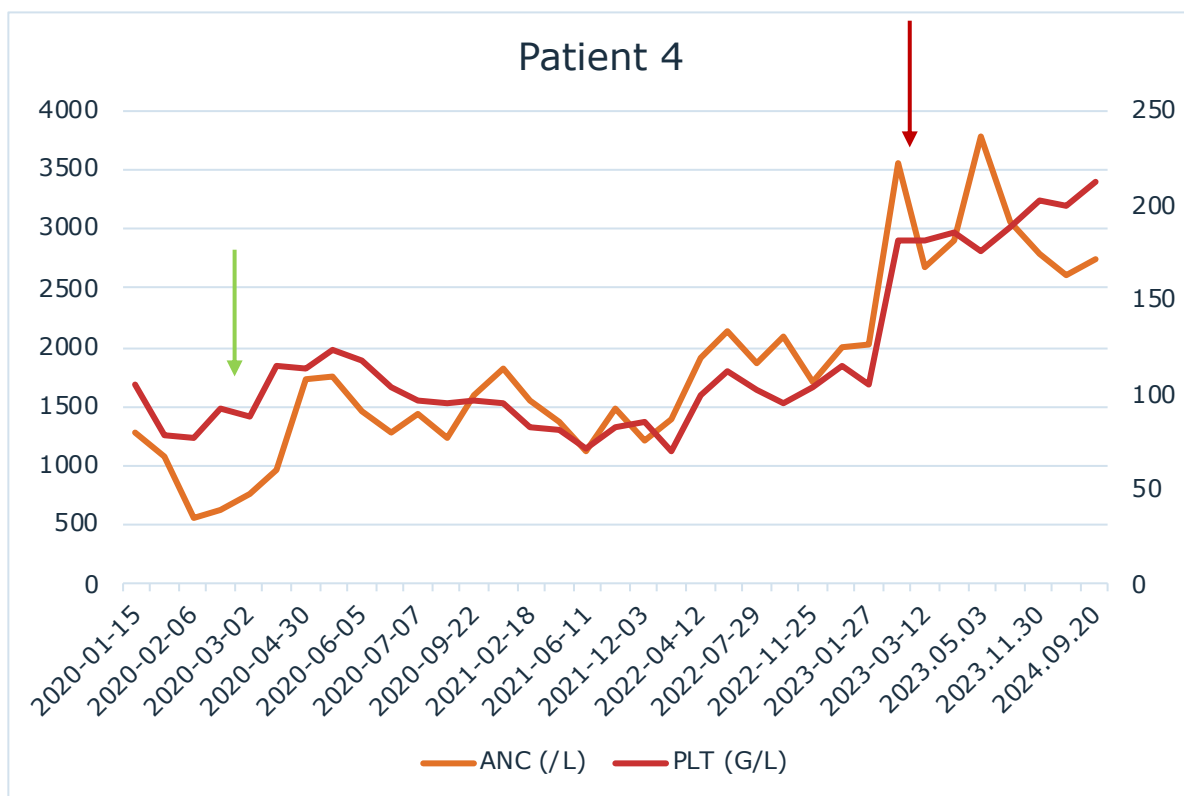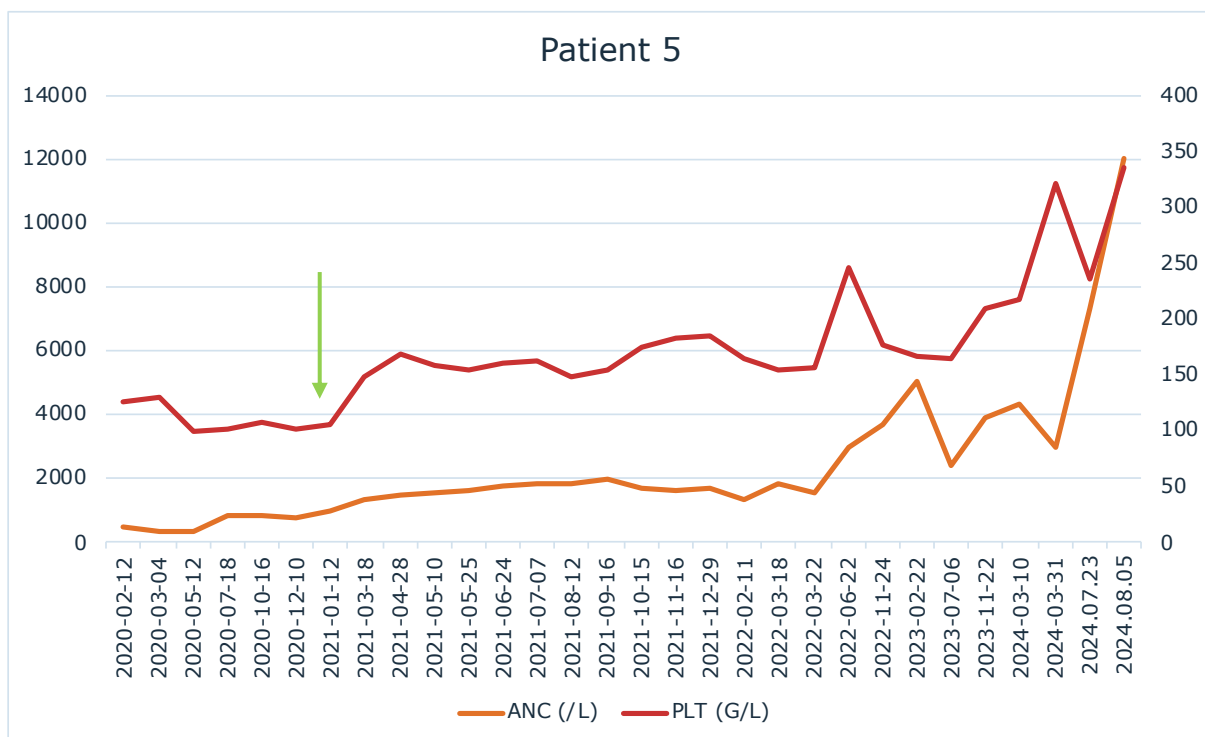

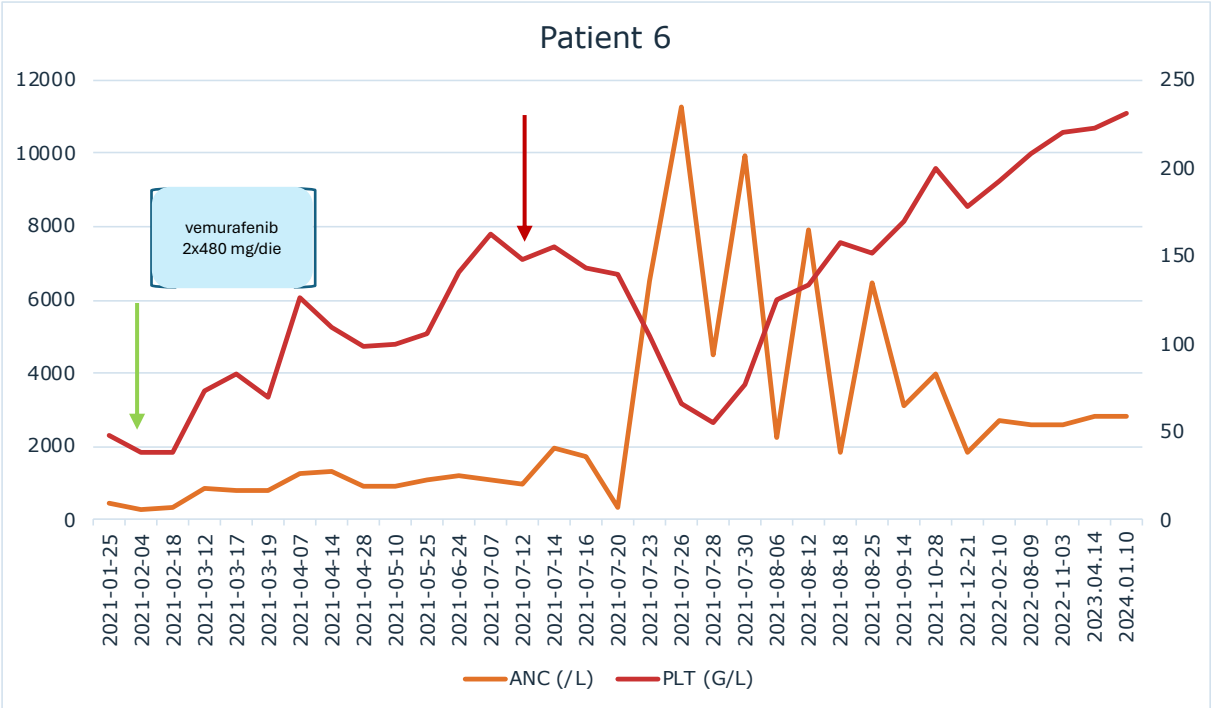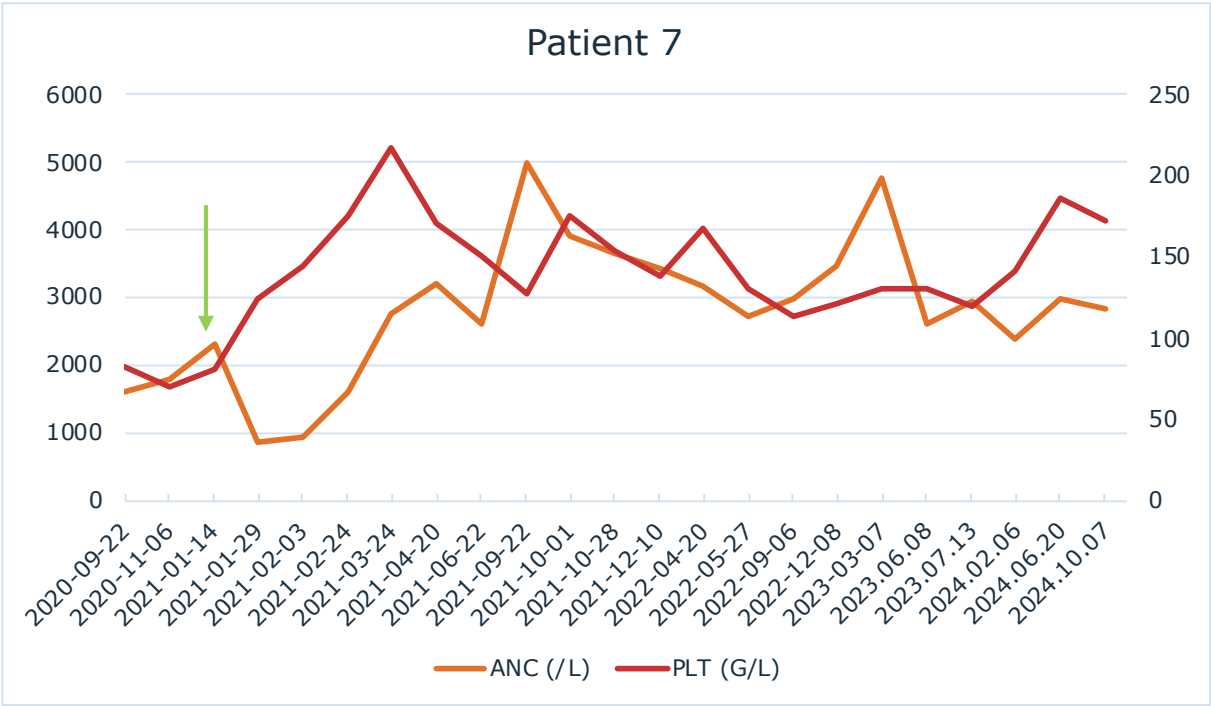

Supplement: Supplementary file 1 [file DataSheet1.pdf]
